# A comparison of mental arithmetic performance in time and frequency domains

**DOI:** 10.3389/fpsyg.2022.921433

**Published:** 2022-09-02

**Authors:** Anmar Abdul-Rahman

**Affiliations:** Department of Ophthalmology, Counties Manukau DHB, Auckland, New Zealand

**Keywords:** wavelet transform, mental arithmetic, ARIMA, GARCH, MODWT, time series

## Abstract

The Heisenberg-Gabor uncertainty principle defines the limits of information resolution in both time and frequency domains. The limit of resolution discloses unique properties of a time series by frequency decomposition. However, classical methods such as Fourier analysis are limited by spectral leakage, particularly in longitudinal data with shifting periodicity or unequal intervals. Wavelet transformation provides a workable compromise by decomposing the signal in both time and frequency through translation and scaling of a basis function followed by correlation or convolution with the original signal. This study aimed to compare the accuracy of predictive models in mental arithmetic in time and frequency domains. Analysis of the author's response time at mental arithmetic using a soroban was modeled for two periods, an initial period (T_*I*_ = 68 days), and a return period (T_*R*_ = 170 days) both separated by an interval of 370 days. The median (min,max) response times in seconds (s) was longer for all tasks during the T_*I*_ compared to the T_*R*_ period (*p* < 0.001), for addition [CT_*Add*_ 62 (45, 127) vs 50 (38, 75) s] and summation [CT_*Sum*_ 68 (47, 108) vs 57(43, 109) s]. Response times were longer for errors regardless of the study period or task. There was an increasing phase difference for the addition and summation tasks during the T_*I*_ period toward the end of the series 49.65^o^ compared to the T_*R*_ period where the phase difference between the two tasks was only 2.05^o^, indicating that both tasks are likely demonstrating similar learning rates during the latter study period. A comparison between time and time/frequency domain forecasts for an additional 100 tasks demonstrated higher accuracy of the maximum overlap discrete wavelet transform (MODWT) model, where the mean absolute percentage error ranged between 5.48 and 8.19% and that for the time domain models [autoregressive integrated moving average (ARIMA), generalized autoregressive conditional heteroscedasticity (GARCH)] was 6.16–10.80%.

## 1. Introduction

Mathematical learning theory was an audacious research concept in learning theory developed in the late 1940s and early 1950s. It attempted to explain fundamental psychological processes through deterministic and probabilistic processes (Atkinson and Calfee, 1963). These processes were then treated analytically to generate precise behavioral predictions for a variety of experimental settings (Howard, 2014). However, the brain has many degrees of freedom at its disposal, therefore deriving correct expressions for physical processes, given only constraints from behavioral data seemed impossible, yet mathematical learning theory has been largely successful in describing essential features of neural data (Howard, 2014).

Time and its influence on neural and psychological data form a central question in many study designs, the analysis follows two distinct yet broadly equivalent modes of modeling information content in time: 1) Time-domain methods, and 2) Frequency-domain methods. Time-domain methods have their origin in the classical theory of correlation. Such methods address the autocovariance and cross-covariance functions of the series. They are described by autoregressive moving-average models for a single series and transfer-function models for two or more causally related series. Frequency-domain analysis represents the signal's energy distribution and includes information on the phase shift, which could be subjected to an inverse transformation to combine all the frequency components and regenerate the signal in the time domain (Pollock et al., 1999a). Frequency-domain methods are based on an extension of the methods of Fourier analysis which originate in the idea that, over a finite interval, any analytic function can be approximated by a weighted sum of sine and cosine functions of harmonically increasing frequencies that are integer multiples of a fundamental frequency (Pollock et al., 1999b). However, Fourier analysis and its probabilities depend on several assumptions these include evenly spaced data of infinite duration with a high sampling rate (Nyquist frequency), Gaussian noise distribution, frequency periodicity, and stationary frequency content of the signal (Solomon, 1991). An additional limitation in modeling time series data is what is known as the Heisenberg-Gabor uncertainty principle, which stipulates that a signal cannot be arbitrarily localized simultaneously both in time and frequency (Flandrin, 1998). Therefore, an inverse relationship exists between the dispersion of a function and the range of the frequencies which are present in its transform (Pollock et al., 1999a). Alfred Haar proposed an alternative system to Fourier analysis in 1909, giving rise to the Haar measure, Haar wavelet, and Haar transform. In his Doctoral thesis appendix in 1910, he described the wavelet transform, and he used the re-scaled square function as an example of an orthogonal function in the theory of series expansion of real functions. These functions are made up of a short positive followed by a short negative pulse (Haar, 1910; Akujuobi, 2022). This method of signal analysis provides a workable compromise in time and frequency resolution arising from the Heisenberg-Gabor uncertainty principle. The means by which wavelets allow signal localization in time and frequency domain simultaneously is by translation of the mother wavelet to obtain time information and scaling the mother wavelet to obtain frequency information (Percival and Walden, 2000b). A wavelet can encapsulate both the trend and cyclical components as well as highlight the intensity of any given point along the time series itself (Bolman and Boucher, 2019). This approach can be applied to a series with non-stationary frequency content, sparse data points, superimposed stochastic processes, trends, breakdown points, discontinuities in higher derivatives, and self-similarity (Bolman and Boucher, 2019; Oliveira et al., 2019). In previous work, an ARIMA model demonstrated favorable forecasting capability in mental arithmetic compared to Wright's model and simple linear regression (Abdul-Rahman, 2020). This study aims to compare the predictive accuracy of time series models of mental arithmetic response times by extension in both time and frequency domains.

## 2. Materials and methods

### 2.1. Mental arithmetic task description

The acquisition of microsurgical skills is dependent on factors other than procedure-related dexterity. Higher cognitive processes determine the procedure's success including error detection, planning, and decision-making (Kohls-Gatzoulis et al., 2004). Additionally, developmental studies demonstrate a correlation between fine motor skills and mathematical ability, suggesting deeper cognitive connections between these tasks (Luo et al., 2007; Fischer et al., 2020). The justification for using mental arithmetic in this study was to model a learning task that can be unambiguously measured for response time, accuracy and long-term memory as a theoretical surrogate for cognitive processes encompassing the aforementioned principles.

A Japanese soroban (abacus) was used to perform the addition and summation of sequential columns of 6-digit numbers. The test consisted of a computer-generated list of 100 digits, ranging between 100,000 and 999,999 (3rd kyu). Test sheets were randomly generated from a website dedicated to training in the use of the soroban (www.sorobanexam.org). In this study, every chain of ten digits is termed a trial and every ten trials are one test. Each test consisted of a chained sequence of 6 addition and 4 summation (addition and subtraction) tests. All tests were conducted at the same time of the day between 7:00 and 7:30 a.m. Two separate computers were used. to administer tests, this was to prevent interference between data collection and the on-screen text-to-speech software used to vocalize the test sheet tasks. One computer used the built-in iOS voice-over application (Big Sur 11.6.1) to vocalize a list of numbers from a test sheet in .pdf format. The computer-generated voice-over reading rate was commenced at 120 words per minute (wpm) at the beginning of the study, which was incrementally increased to 200 wpm toward the end of the study. Increments in reading rates were adjusted at intervals dependent on task response time to minimize auditory comprehension errors. The second computer (Big Sur 11.6.1) was used to execute custom software written in R computing language (R Core Team, 2020), which captured task response time in seconds (s). The data capture program was executed simultaneously with the on-screen voice-over application. The software consisted of a for-loop that recorded the loop running time. When the 10 digits/trial column computation was completed, the timer loop displayed an on-screen text prompt to finalize the outcome of the computation as y/n for correct/errors, respectively. Outcomes were recorded as errors when the number configured by the soroban beads did not match the pre-printed trial column result. For each test captured data including date, time, test type, response time, and trial outcomes, these were automatically appended to a locally stored file in .csv format for further analysis. For each test, no breaks were provided between sequential trials of 10-digit columns. The test was concluded when 10 trial columns were computed. The total test duration, therefore, ranged from 7 to 12 min. Tests that were interrupted by errors in the text-to-speech vocalization were considered outliers and were excluded from the analysis. The cumulative calculation time was defined as response time (s) for the addition (CT_*Add*_) or summation (CT_*Sum*_) tasks for an individual study period regardless of the trial outcome. Details of the principles of complementary arithmetic and the principle of calculation using the soroban are discussed in previous work (Abdul-Rahman, 2020).

The total study duration was 608 days divided in two periods: an initial period (T_*I*_) lasting 68 days (1,610 trials, total test time 29.38 h), and a return period (T|_*R*_) for 170 days (1,700 trials, total test time 25.35 h), both separated by an interval of 370 days. This was to assess whether the skills in soroban-based calculation degraded over a period where the task was not practiced. The time series sample size (N) for T_*I*_ and T_*R*_ study periods was *N* = 947, 1,020 for addition, and *N* = 663, 680 for summation, respectively. Response time, error percentage and long-term performance retention were used to represent learning gains, accuracy, and long-term memory, respectively.

### 2.2. Statistical analysis

Distributions of CT_Add_ and CT_Sum_ were modeled using fitdistplus() package (Delignette-Muller and Dutang, 2015). Since distributions of response times were non-normal, standard performance indices included response time (performance gain) expressed as the median, and the interquartile range (IQR), mean and standard error were used where appropriate. Error percentage indicated the rate of incorrect trial responses, and served as a measure of response accuracy (Steinborn and Huestegge, 2016). Pearson's Chi-squared test (χ^2^) with Yates' continuity correction was used to evaluate the accuracy of addition and summation tasks in a contingency table. The effect size was calculated using the Cramer *V*-test (Cohen, 2013), using the following scale: small 0.10–0.30, moderate 0.30–0.50, large ≥ 0.50. Pairwise analysis of variance (ANOVA) was used for hypothesis tests of response time. Mosiac plots were used to visualize the hypothesis test outcomes. A *p* < 0.05 was considered statistically significant for all tests.

### 2.3. Analysis in the time domain

Time series models were used to evaluate mean response time (±) standard error (se). Model predictive accuracy was determined by comparing the lowest mean absolute percentage error (MAPE). Analysis in the time domain was achieved using autoregressive integrated moving average models (ARIMA) for the T_*I*_ period. However, due to autoregressive conditional heteroscedasticity (ARCH) effects generalized autoregressive conditional heteroscedasticity models (GARCH) were used for the T_*R*_ period.

#### 2.3.1. Autoregressive integrated moving average models

An ARIMA time series model is defined by three terms (p,d,q), which represent the autoregressive (p), integrative (d), and the moving average (q) parameters of the model. Iterative tests of the ARIMA model (p,d,q) order were done using auto.arima() command from the R forecast package for the T_*I*_ period time series. This command combines unit root tests, minimization of the corrected Akaike's Information Criterion (AICc), and Maximum likelihood estimation (MLE) to obtain the optimum model fit (Hyndman and Athanasopoulos, 2018). Model order validity was confirmed by plotting the autocorrelation (acf) and partial autocorrelation (pacf) functions. Curve fitting diagnostics were undertaken both numerically selecting the model demonstrating the lowest Akaike information criterion amongst the compared ARIMA models of different orders using the lowest MAPE value. The characteristic roots of the time series equations were plotted to assess whether the model is close to invertibility or stationarity in relation to the complex unit circle, where roots close to the unit circle may be considered numerically unstable. After visual inspection of the time series plot for stationarity, the assumption was confirmed by applying two statistical tests: the augmented Dickey-Fuller test, where a lag length (k) was chosen by default for this test (CT_*Add*_ *k* = 7, and CT_*Sum*_ *k* = 6), where a *p* *leq* 0.01 was considered statistically significant for all four mental arithmetic tasks. The Kwiatkowski-Phillips-Schmidt-Shin test (KPSS) was then applied, which is used for testing the null hypothesis that an observable time series is stationary around a deterministic trend, or is non-stationary due to a unit root. This test demonstrated a statistic of < 1% of critical value for all tasks, confirming stationarity after differencing, which is in turn a critical step for further statistical validity. Both the acf plot of the residuals and the Ljung-Box test were performed to assess for autocorrelation within the series. Autoregressive conditional heteroscedasticity (ARCH) among the lags was assessed using the McLeod-Li test, where the T_*I*_ models showed that ARCH effects were absent. However, the T_*R*_ period demonstrated significant ARCH effects. From a total of 30 lags, there was 100% heteroscedastic error (lag 1 to 30) in the addition series and 23% in the summation series (lag 7–30). Therefore a GARCH model was used to fit the time series for the T_*R*_ period.

#### 2.3.2. Generalized autoregressive conditional heterosedasticity models

Conditional heteroscedasticity implies that there is a non-constant variance of the predictors in the time series. The GARCH-in-mean (GARCH-M) model was used to represent the T_*R*_ time series tasks. Skewed student-t distribution was used to model the residuals. Model fit was assessed using information criteria, Ljung-Box, and Pearson's goodness of fit tests. Forecast accuracy parameters were calculated by splitting the T_*R*_ period time series to training and test sets addition (training = 900, test = 180 data points), and summation (training = 500, test = 120 data points). Bootstrapping was used to generate forecasted data (100 data points) from the GARCH models.

### 2.4. Analysis in the time and frequency domains

Models of response time in the frequency domain were generated using both continuous and maximal overlap discrete wavelet transform (MODWT) as detailed below. The latter model was used to generate the time series forecast.

#### 2.4.1. Wavelet transform

The cyclical component of a time series, which is defined as (regular or periodic) oscillations around the trend that remove the irregular component and depict the series as an expansion and contraction phase, is quantified by frequency domain analysis. The choice of wavelet depends upon the type of signal to be analyzed and the application, therefore there is no absolute way to choose a certain wavelet from the extended family of wavelet basis functions (Mallat, 1999; Fugal, 2009; Haddadi et al., 2014). Two transformations were applied to each time series, a continuous (CWT) and discrete (DWT) wavelet transform. The first applied transformation was a Morlet wavelet transform (continuous wavelet) using the WaveletComp package. The purpose was to estimate the phase difference between the tasks for each study period (Roesch and Schmidbauer, 2018). Pre-processing and parameter selection for the data before applying the transformation included removing 10 data points at the extremes of each time series to reduce the edge effect. As there were 10 trials per test the time resolution (dt) = 10 was selected combined with a high-frequency resolution (1/250). The phase of a given time series can be viewed as the position in the pseudo-cycle of the series and it is parameterized in radians ranging from −π to π (Cazelles et al., 2007). MODWT is a modified version of the discrete wavelet transform (DWT), it allows to perform a multi-resolution analysis which is a scale-based additive decomposition particularly important for a longitudinal data with multiple frequency content, additionally, unlike a DWT it is insensitive to the choice of the starting point for the series in its decomposition (Percival and Walden, 2000a; Zhu et al., 2014).

## 3. Results

### 3.1. Task outcomes

**Figure 1:**
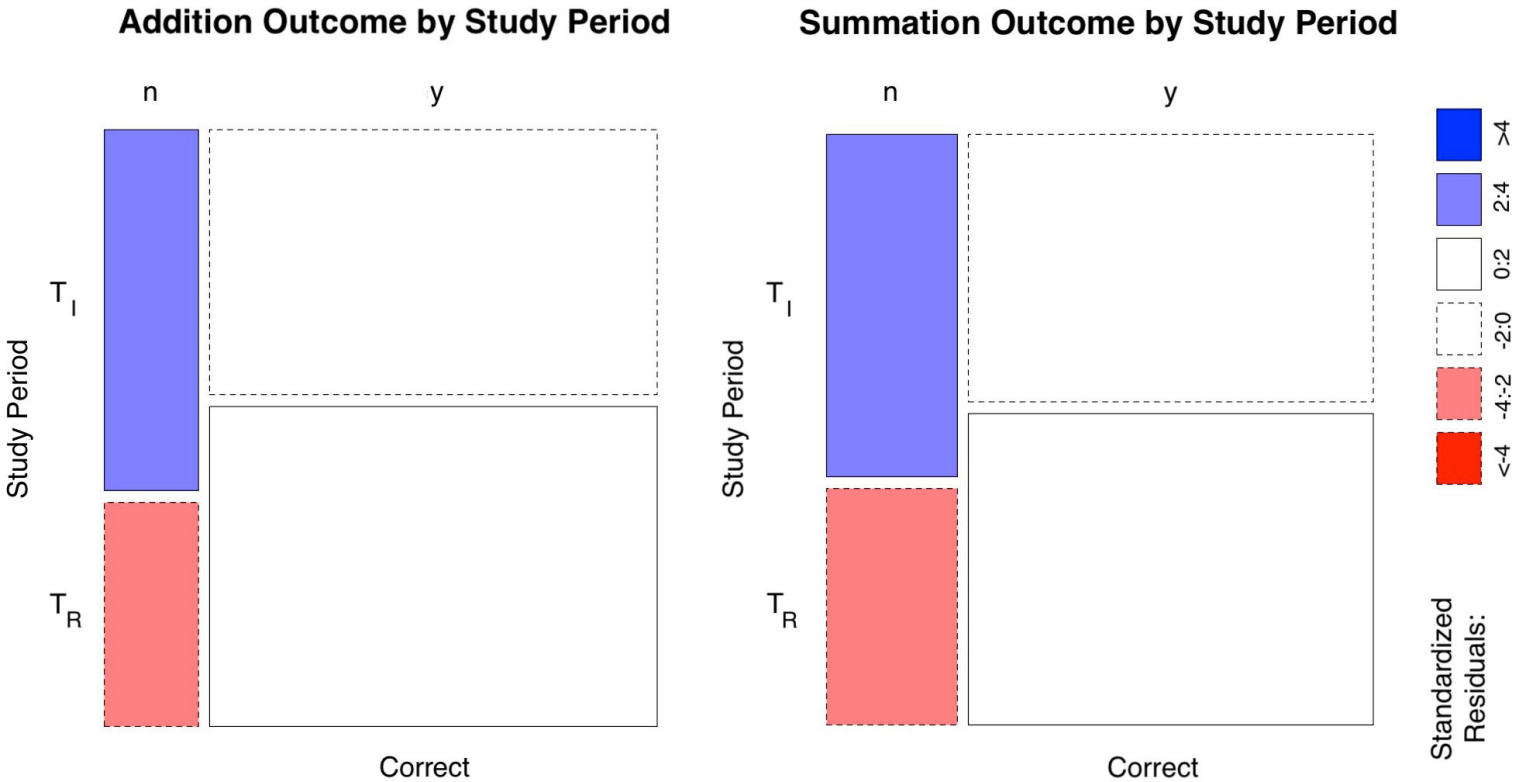
Mosaic plots of the mental arithmetic learning task outcomes subsetted by study phase. The width of the columns indicates the proportions in each group, which corresponds to the column totals in Table 2. The height of the boxes (rows/horizontal break) is the proportions of the outcomes in each of the initial (T_*I*_) and return (T_*R*_) study periods, which represents the rows of Table 2. Standardized residuals indicate that there are more (blue) observations than would be expected under the null model for erroneous responses in the T_*I*_ period and less (red) in the T_*R*_ period for both tasks. Since the horizontal bars are at unequal levels, therefore there is a statistically significant difference between task outcome and study period. i.e. there was a statistically significant difference between correct responses and errors in both the T_*I*_ and T_*R*_ study periods. y=yes, n = no.

Total task response time for the T_*I*_ period was longer despite the lower number of tasks in this period, where a total of 1,610 trials (test time of 29.38 h) were performed, compared to the T_*R*_ period where a total of 1,700 trials over a shorter total test time of 25.35 h was undertaken. Tables 1, 2 summarize the response times and outcomes for the tasks by study period. There was a statistically significant (*p* < 0.0001) improvement in overall task accuracy 74.8 vs. 84.4% for T_*I*_ and T_*R*_ periods, respectively, (χ^2^ = 46.29, *N* = 3,310, df = 1), here the effect size was small (Cramer *V* = 0.12). Highest improvement occurred with addition where the number of correct responses increased from (T_*I*_ = 45.7% to T_*R*_ = 52.3%) compared to the correct responses in summation (T_*I*_ =29.1% to T_*R*_ = 32.1%). Mosaic plots (Figure 1) provide a summary of the hypothesis tests for task outcomes by study period.

**Table 1:** Response time for addition and summation tasks for the initial and return study periods.

**Learning period = 68 days = 29.38 h**					
**Task**	**Median (IQR)**	**Min**	**Max**	**Skew**	**Kurtosis**
Addition	62 (12)	45	127	1.268	3.81
Summation	68 (16)	47	108	0.831	0.439
**Interval = 370 days**					
**Return period = 170 days = 25.35 h**					
**Task**	**Median (IQR)**	**Min**	**Max**	**Skew**	**Kurtosis**
Addition	50 (6.25)	38	75	0.905	1.231
Summation	57 (9)	43	109	1.204	3.72

Response time median and interquartile range (IQR) for the cumulative time for addition and summation tasks. Max, maximum; Min, minimum.

**Table 2:** Response time for addition and summation tasks subsetted by test outcomes.

**Learning period = 68 days = 29.38 h**					
**Task**	**RT_*c*_**	**Correct (n%)**	**RT_*e*_**	**Errors (n%)**	**Total**
Addition	61 (11)	736 (77.7%)	66 (14)	211 (22.3%)	947 (58.8%)
Summation	66 (15)	469 (70.7%)	71 (16)	194 (29.3%)	663 (41.2%)
Total		1205 (74.8%)		405 (25.2%)	1610
**Interval = 370 days**					
**Return period = 170 days = 25.35 h**					
**Task**	**RT**_*c*_	**Correct (n%)**	**RT**_*e*_	**Errors (n%)**	**Total**
Addition	50 (7)	889 (87.2%)	52 (6.5)	131 (12.8%)	1020 (60.0%)
Summation	56 (9)	546 (80.3%)	58 (10)	134 (19.7%)	680 (40.0%)
Total		1435 (84.4%)		265 (15.6%)	1700

Mental arithmetic tasks were performed over 608 days, which consisted of a learning followed by a return period after an interval of 370 days. There was improvement in task response time especially for addition in the T_R_ study period. RT = median (interquartile range) response time in seconds for correct responses (RT_c_) and errors (RT_e_). Percentages are calculated for the table rows.

### 3.2. Task performance time

**Figure 2:**
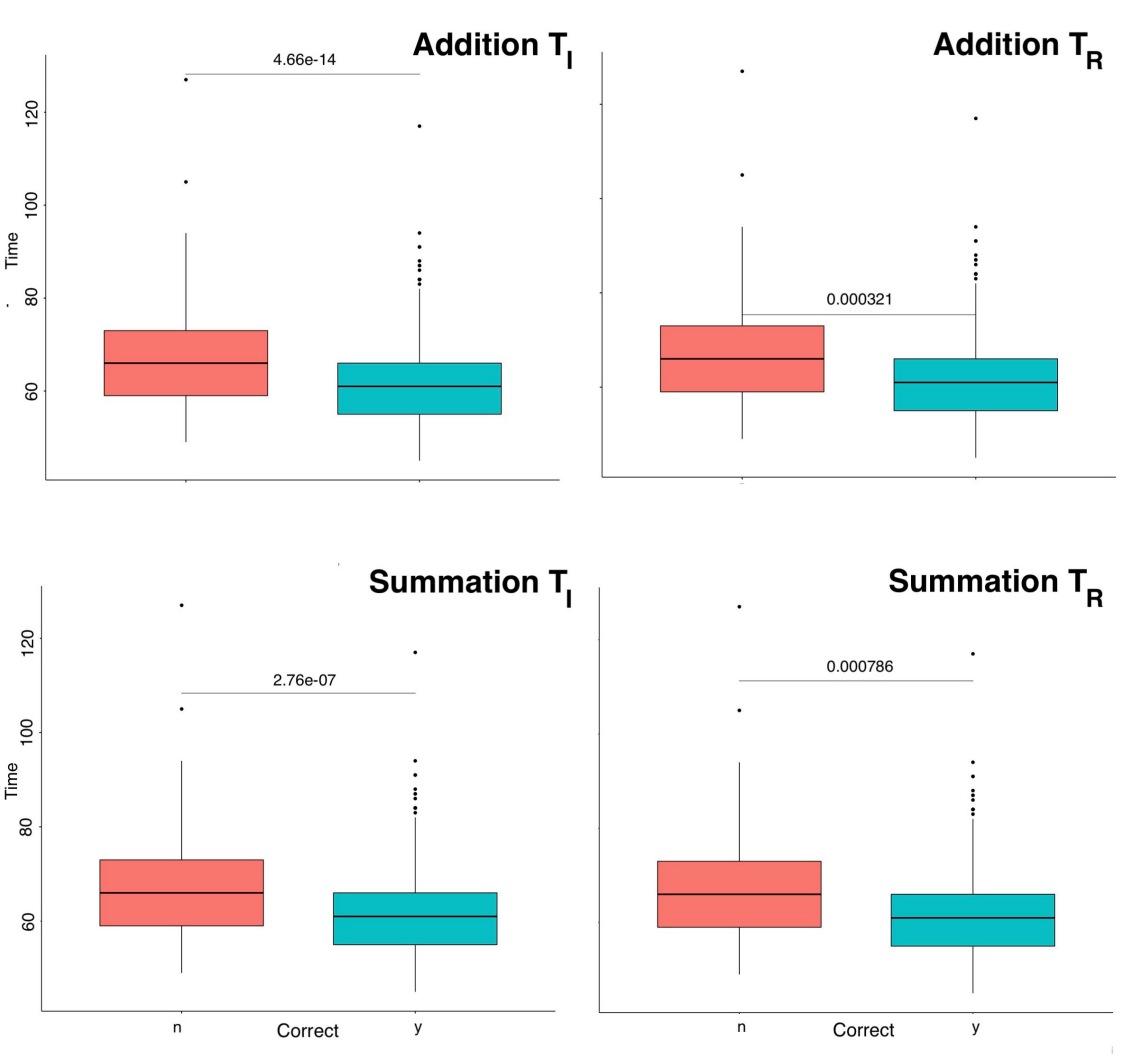
Boxplot of mental arithmetic response time subsetted by outcome. Response times for errors were consistently longer than correct responses for both tasks. Differences were statistically significant for all study periods (*p* < 0.001). T_*I*_ = initial study period, T_*R*_ = return study period, Time= task response time, y=yes, n=no.

The distribution of performance times (CT_Add_ and CT_Sum_) were non-normal. The cumulative distribution functions demonstrated a lognormal fit with a skew to the right for CT_Add_ and CT_Sum_ for all study periods. Table 1 summarizes the descriptive statistical parameters for the response time, where it can be noted that addition tasks had consistently shorter response times than summation. Within task group (addition, summation) pairwise ANOVA (Figure 2) showed a statistically significant difference between response times subsetted by study period (Table 2) with erroneous responses being longer than correct responses during all study periods regardless of the task category (*p* < 0.001). To evaluate the influence of the interval where no practice was undertaken on the response time, the mean response time for the last and first 20% of trials for the T|_*I*_ and T_*R*_ periods were compared, these, respectively, were (54.27, 57.79 s) for addition and (59.49, 65.75) for summation, these differences were statistically significant (*p* < 0.0001), that indicated some degradation of performance.

### 3.3. Analysis in the time domain

The sub-daily time series frequency ranged from 10 to 70 trials (median 30) per day for the T_*I*_ period and was more consistent at 10 trials/day for the T_*R*_ period.

#### 3.3.1. Autoregressive integrated moving average model

**Figure 3:**
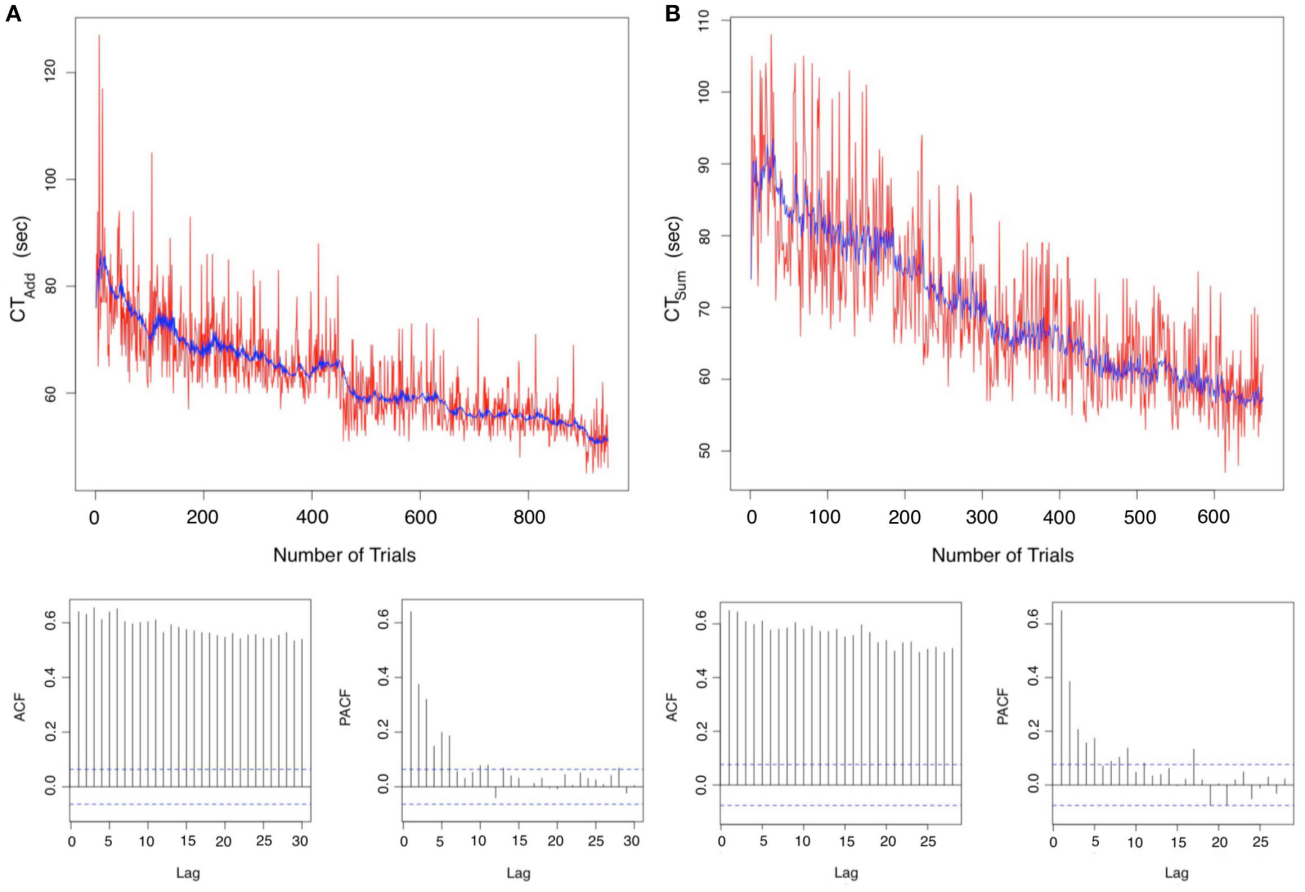
Analysis in the time domain for the initial period data: Autoregressive integrated moving average (ARIMA) model for the **(A)** Addition task time Series ARIMA (2,1,3) with Drift **(B)** Summation time series plot ARIMA (4,1,3) with Drift. Autocorrelation defines the degree of similarity between a particular time series and a lagged version of itself over subsequent time intervals. The autocorrelation functions (ACF) demonstrate a declining geometric pattern consistent with the trend of the time series of accelerating response time, which remain significant at the 95% confidence interval for a lag >25. Partial autocorrelation functions (PACF) show that lags <6 and <5 are significant for addition and summation, respectively. Consequently, confirming an autoregressive model fit below the 5th order for both tasks.

Figures 3A,B are time series plots for the T_*I*_ addition and summation tasks, respectively, with autocorrelation (acf) and partial autocorrelation (pacf) correlograms. In these graphs the ARIMA model fit is represented by the central blue line. The acf plots show a geometric pattern consistent with a declining trend over time. Additionally, a strong correlation of the sequential points up to lag 30 can be noted. The pacf shows a significant correlation at the 95% confidence interval up to lag 6 for addition and lag 5 for summation. These tests confirm the learning gains with a <20% correlation of test scores at each 6th trial in the series for addition and 5th trial for summation at the 95% confidence interval. A favorable model fit in the time series can be visually confirmed as a blue line in the time series plots. Model coefficient and fit parameters are listed in Table 3 where it can be noted that MAPE was 6.16 and 7.94% for CT_*Add*_ and CT_*Sum*_, respectively. The ARIMA equations can be written in the standard form:

Addition task time Series ARIMA (2,1,3) with Drift for the initial learning period:


(1) $$\begin{array}{l} \left(1-\phi_{1}B-\phi_{2}B^{2}\right)\;\left(1-B\right)\left(y_{t}-\delta t\right)=\left(1+\theta_{1}B+\theta_{2}B^{2}+\theta_{3}B^{3}\right)\;\varepsilon_{t} \end{array}$$

Summation time series ARIMA (4,1,3) with Drift for the initial learning period:


(2) $$\begin{array}{l} \begin{array}{l} \left(1-\phi_{1}B-\phi_{2}B^{2}-\phi_{3}B^{3}-\phi_{4}B^{4}\right)\;\left(1-B\right)\left(y_{t}-\delta t\right)\\ =\left(1+\theta_{1}B+\theta_{2}B^{2}+\theta_{3}B^{3}\right)\;\varepsilon_{t} \end{array} \end{array}$$

**Table 3:** Autoregressive integrated moving average model parameters for mental arithmetic addition and summation tasks during the initial study period.

**Addition ARIMA (2,1,3) with drift**	**ar1**	**ar2**	**ma1**	**ma2**	**ma3**	**drift**		
Coefficients	–1.4012	–0.8829	0.4615	–0.4722	–0.8061	–0.0324		
se	0.0716	0.1214	0.0734	0.1050	0.1165	0.0104		
*p*-value	<0.0001	<0.0001	<0.0001	<0.0001	<0.0001	<0.002		
AIC = 5974.74, RMSE = 5.64, MAPE = 6.16								
**Summation ARIMA (4,1,3) with drift**	**ar1**	**ar2**	**ar3**	**ar4**	**ma1**	**ma2**	**ma3**	**drift**
Coefficients	–0.9106	–0.7419	0.1901	0.0643	0.0382	–0.0347	–0.9313	–0.0459
se	0.0550	0.0651	0.0589	0.0424	0.0380	0.0454	0.0341	0.0088
*p*-value	<0.0001	<0.0001	<0.001	<0.12	<0.31	<0.46	<0.0001	<0.0001
AIC = 4491.29, RMSE = 7.08, MAPE = 7.94								

Addition and summation time series required a 2nd and 4th order autoregressive terms. ar, autoregressive coefficients; ma, moving average coefficients; se, standard error; RMSE, root mean square error; MAPE, mean absolute percentage error; AIC, Akaike Information Criterion.

#### 3.3.2. Generalized autoregressive conditional heterosecdasticity

**Figure 4:**
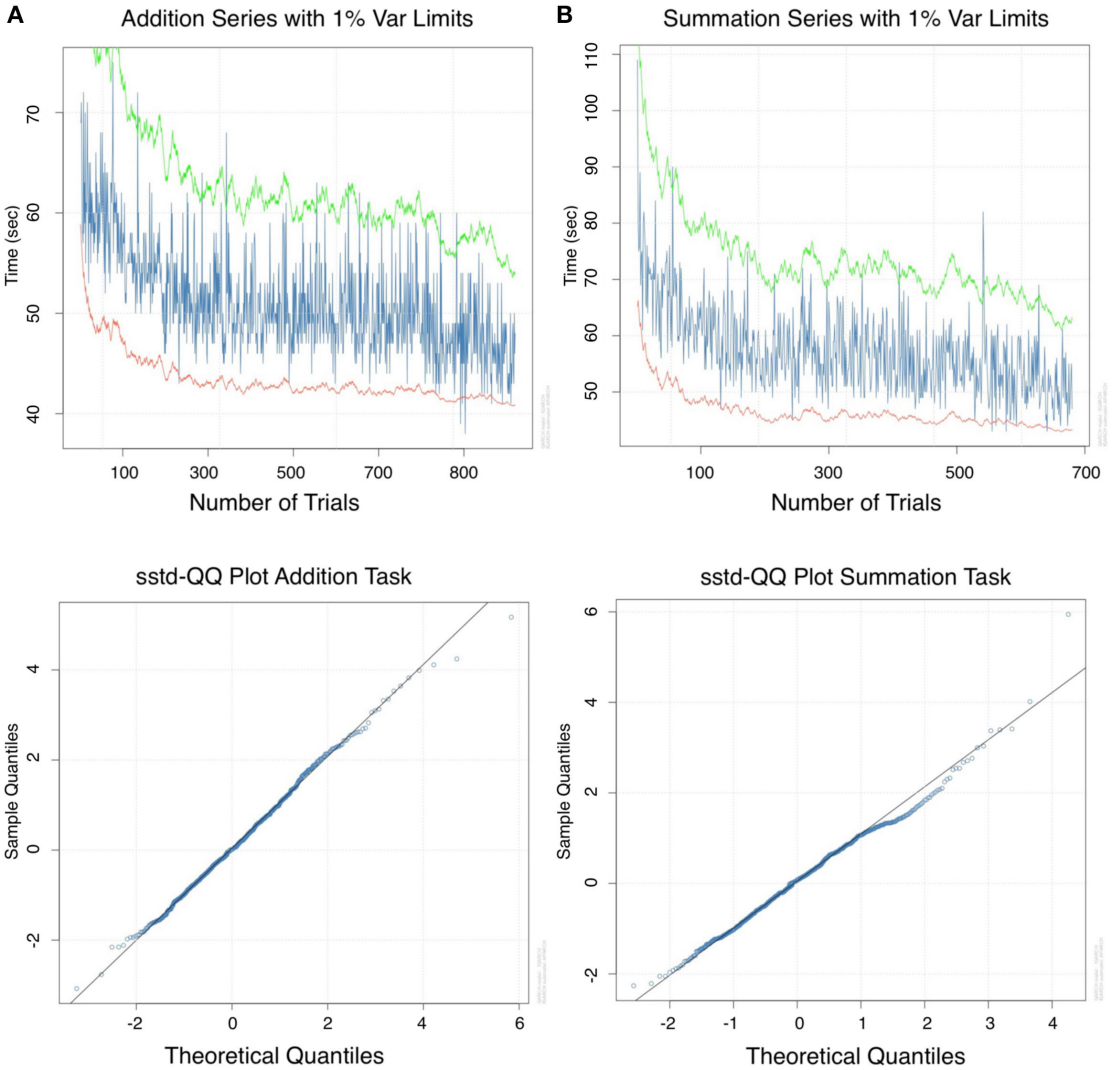
Analysis in the time domain for return period data: Generalized autoregressive conditional heteroscedasticity (GARCH) model for **(A)** addition and **(B)** for summation with 1% variance lines. A GARCH model was required for this period due to volatility in the series, this was likely due to the number of complementary operations in a single calculation, a variable unaccounted for in the model. QQ plots of the standard residuals for both tasks demonstrated a favorable model fit.

As the T_*R*_ period demonstrated significant ARCH effects. From a total of 30 lags, there was 100% heteroscedastic error (lag 1 to 30) in the addition and 23% in the summation series (lag 7–30). Therefore a GARCH model was fitted to the time series of the T_*R*_ period. Figures 4A,B are GARCH model fit plots with 1% variance. Model goodness of fit for the addition and summation tasks is demonstrated graphically using QQ plots in Figures 4A,B, respectively. The equation coefficients (Table 4) achieved statistical significance (*p* < 0.001) for both tasks. Adjusted Pearson goodness of fit test demonstrated *p* > 0.05 for both models. The GARCH model predicted a mean of 49.48 ± 3.46 and 56.49 ± 5.39 s MAPE 8.03 and 10.80% for CT_*Add*_ and CT_*Sum*_, respectively. The equations of the GARCH models fitted with a skewed student-t distribution of the standardized residuals can be written in the form:

GARCH addition in the T_*R*_ period:


(3) $$\begin{array}{c} \begin{array}{c} x_{t}=42.00+0.08\sigma_{t}+\epsilon_{t}\\ \sigma_{t}^{2}=0.01+0.003\cdot \epsilon_{t-1}^{2}+0.99\cdot \sigma_{t-1}^{2}\\ \epsilon_{t}=\sigma_{t}\cdot \left(-1.00\right) \end{array} \end{array}$$

GARCH summation in the T_*R*_ period:


(4) $$\begin{array}{c} \begin{array}{c} x_{t}=44.78+0.08\sigma_{t}+\epsilon_{t}\\ \sigma_{t}^{2}=0.02+0.01\cdot \epsilon_{t-1}^{2}+0.98\cdot \sigma_{t-1}^{2}\\ \epsilon_{t}=\sigma_{t}\cdot \left(-1.00\right) \end{array} \end{array}$$

**Table 4:** Generalized autoregressive conditional heteroscedasticity model coefficients for mental arithmetic addition and summation tasks for the return study period.

**Coefficients**	**Addition**	**se**	**Summation**	**se**
Mu (μ)	42.001325	0.084257	44.780064	0.225086
Gamma (γ)	0.568904	0.003714	0.367082	0.003766
Omega (ω)	0.006396	0.000034	0.016443	0.000077
Alpha (α1)	0.003531	0.000057	0.005856	0.000017
Beta (β1)	0.991902	0.000001	0.981010	0.00
Eta (η11)	–1.000000	0.000005	–0.999546	0.001007
Lambda (λ)	0.078881	0.002103	0.076132	0.001048
Skew	1.424227	0.058776	1.482621	0.114878
Shape	6.679549	0.264986	19.469519	0.468663
Log likelihood	–2768.72		–2117.72	
AIC	5.45		6.26	
BIC	5.49		6.31	

The model shows a positive skewed distribution as the skew is >1 (addition = 1.424227, summation = 1.482621). Therefore skewed standard student-t distribution was used to fit the residuals. Shape is based on degrees of freedom higher values (addition = 6.679549, summation = 19.469519) indicate vertical tails. The p < 0.001 for all coefficients.

### 3.4. Time and frequency domain analysis

#### 3.4.1. Continuous morlet wavelet transform

In the T_*I*_ period, the maximum power was 101.905 and a steep power gradient was distributed throughout the addition task series transform. The power declined to a maximum of 27.681 during the T_*R*_ period in this series. The power spectrum for the summation task during the T_*I*_ period was at a maximum of 25.424, and during the T_*R*_ period a maximum of 30.402 was attained. Therefore the difference in maximum power between study periods was higher for addition (74.224) compared to summation (–4.978) consistent with the higher learning gains in this task.

**Figure 5:**
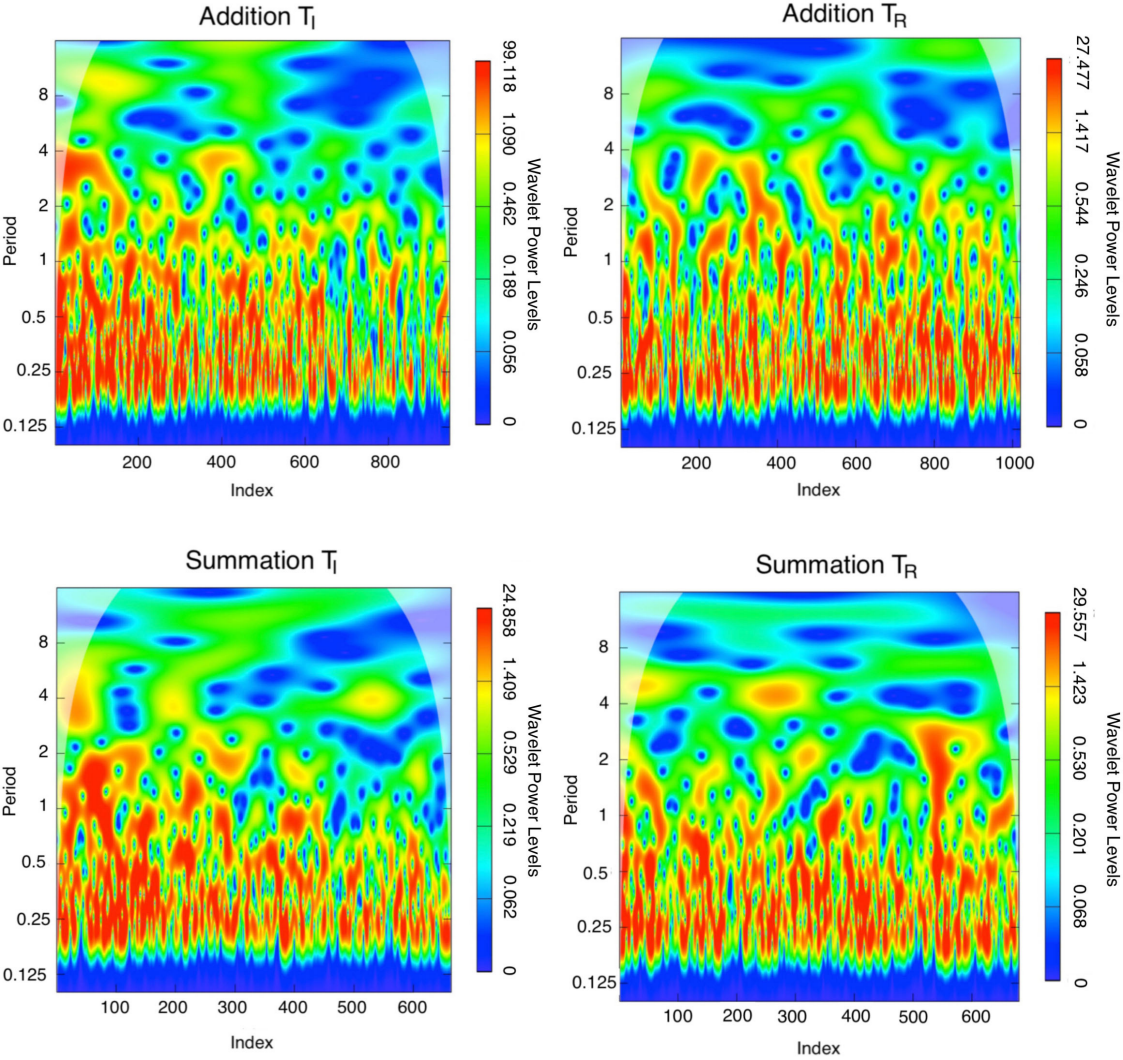
Morlet continuous wavelet transform for the addition and summation tasks for the initial study and return study periods, which highlights the wavelet spectra in each series. The colors code for wavelet power values from dark blue (low values), to red (high values). The low contrast zone in the image periphery indicates the cone of influence that delimits the region not influenced by edge effects. The wavelet power spectrum depicts the evolution of a time series' variance at various frequencies, with periods of high variance corresponding to periods of high power at various scales. Whereas the highest wavelet power is distributed irregularly throughout the T_*R*_ series, wavelet power is highest at index < 200 for the T_*I*_ series especially for the addition task. These findings quantify the accelerated response time in the earlier part of the T_*I*_ series and the more consistent response time in the T_*R*_ series with interspersed volatility. sstd= skewed student-t distribution.

**Figure 6:**
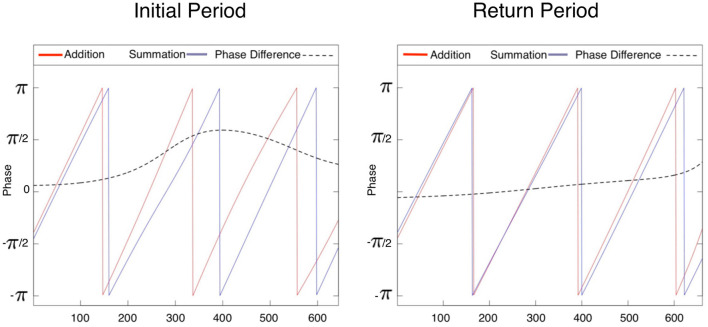
Within study period phase of the two tasks computed in the first 600 data points in the time series. Whereas a higher phase difference [mean(min, max)] in the initial study period 0.3336π(0.0612π, 0.5927π) indicates a that the series are non-synchronized initially, in the return study period the reduction of the phase difference to 0.0507π(-0.0561π, 0.2873π) suggests that displacements of periodic phenomena in two time series are minimally different. This likely indicates similar learning rates for both tasks in the latter study period.

Continuous wavelet transforms are demonstrated in Figure 5, where the number of trials of the series is displayed on the horizontal axis, while the vertical axis shows the scale (the lower the frequency, the higher the scale). Colors toward the red end of the scale represent regions with significant power, while colors toward the blue end (cold regions) of the scale signify lower power between the series. Cold regions beyond the significant areas represent time and frequencies with no dependence in the series. It can be observed that a dominant high power spectrum occurs early in the T_*I*_ period, whereas in the T_*R*_ period high power and volatility were distributed throughout the series. The phase difference mean (min, max) between tasks (addition and summation) within a study period is demonstrated in Figure 6. Whereas, during the T_*I*_ period there was an increasing phase difference toward the end of the series 0.3336π (0.0612π, 0.5927π) [60.04^o^(11.02^o^, 106.69^o^)], the phase of summation task preceded that of addition throughout the series, in contrast during the T_*R*_ period the phase difference between the two tasks was minimal 0.0507π (–0.0561π, 0.2873π) [9.12^o^(-10.09^o^, 51.71^o^)].

#### 3.4.2. Maximum overlap discrete wavelet transform

**Figure 7:**
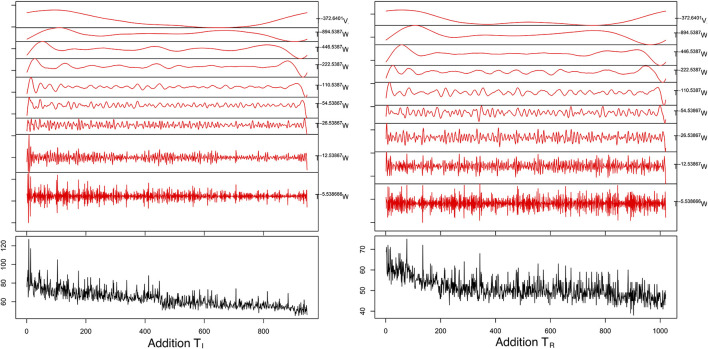
Graphical representation of maximal overlap discrete wavelet transform (red plot) for the addition tasks (black plot). The decomposition was done using the using a d1-8 Daubechies wavelet, where W and V are the coefficient vectors that have been circularly advanced by the exponent in the shift operator (T).

**Figure 8:**
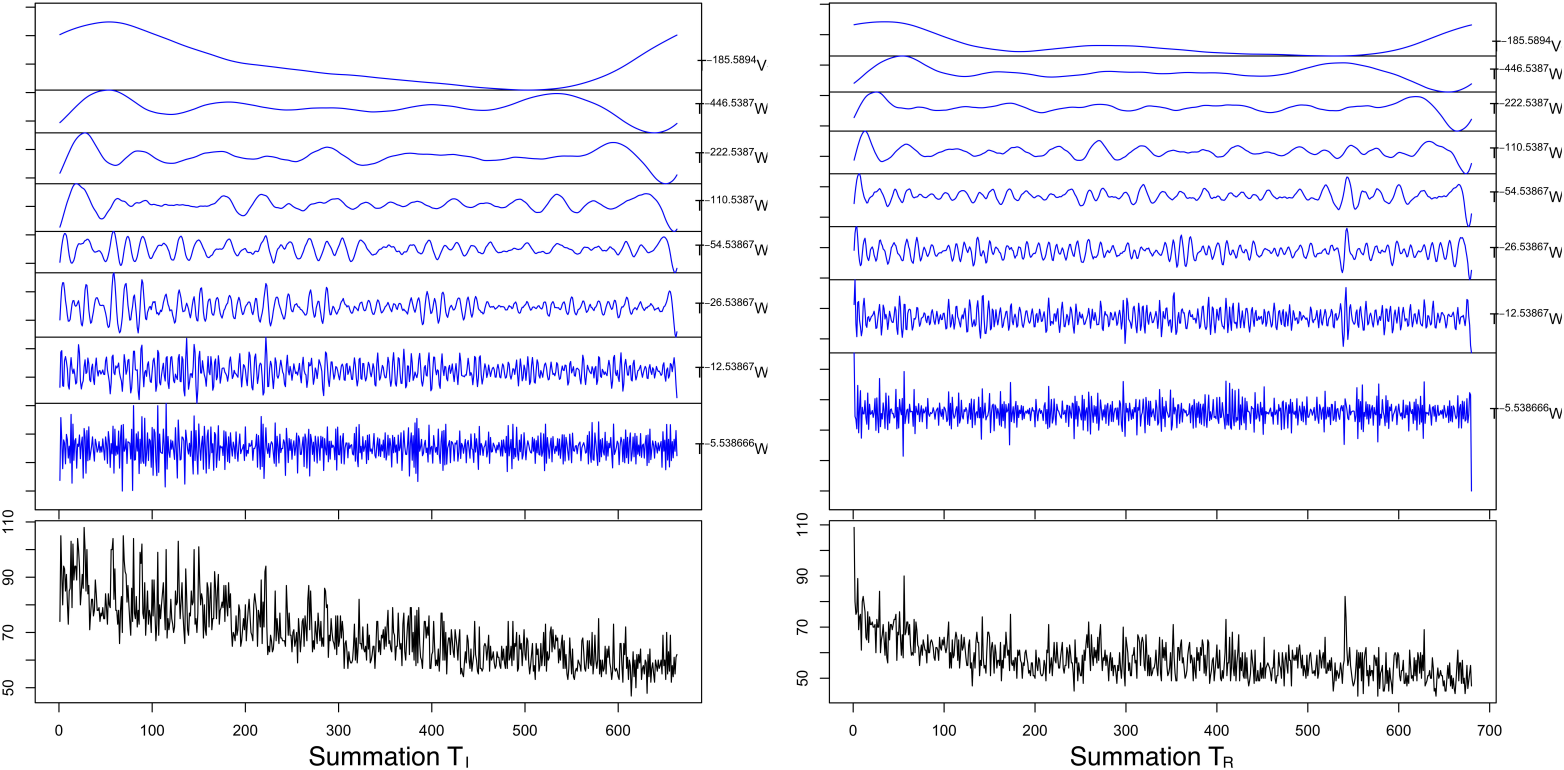
Graphical representation of maximal overlap discrete wavelet transform (blue plot) for the summation tasks (black plot). The decomposition was done using the using a d1-8 Daubechies wavelet, where W and V are the coefficient vectors that have been circularly advanced by the exponent in the shift operator (T).

Maximum Overlap Discrete Wavelet Transform demonstrated that 99.063–99.573% of the series energy was represented in the d1–d3 Daubechies waveforms (Table 5) for all four series. For the T_*I*_ period the MODWT model predicted a reduction of both CT_*Add*_ to a mean of 51.36 ± 0.12 s with an error of 6.07% and CT_*Sum*_ to a mean of 56.76 ± 0.10 s with an error of 6.85% over 100 forecasted trials. For the T_*R*_ period MODWT predicted a mean of 44.63 ± 0.07 and 53.35 ± 0.11 s MAPE 5.48 and 8.19% over 100 forecasted trials for CT_*Add*_ and CT_*Sum*_, respectively. Forecasted mean response time and accuracy for the time and time/frequency domain models are compared in Table 6 and demonstrated graphically in Figures 7, 8.

**Table 5:** Energy for Daubechies wavelet coefficients (d1-d9) are calculated in percentages, s9 is the Maximum overlap discrete wavelet transform scaling coefficient.

	**Addition T_I_**	**Addition T_R_**	**Summation T_I_**	**Summation T_R_**
d1	80.482	81.024	79.023	76.468
d2	16.719	15.772	16.945	19.393
d3	2.372	2.653	3.377	3.202
d4	0.350	0.465	0.535	0.601
d5	0.064	0.070	0.083	0.182
d6	0.009	0.010	0.027	0.070
d7	0.002	0.001	0.007	0.033
d8	0.000	0.000	0.002	0.020
d9	0.000	0.001	0.002	0.018
s9	0.002	0.002	0.000	0.015

Noted is that >90% of the energy of all time series is encapsulated in the first two wavelet decompositions.

**Table 6:** Mean mental arithmetic response times (s) forecasted for 100 trials using time (ARIMA, GARCH) and time/frequency (MODWT) domain model fits.

**Task**	**Mean**	**ARIMA**	**GARCH**	**MODWT**
Addition T_I_	62.92 ± 0.30	49.50 ± 0.10 (6.16%)		51.36 ± 0.12 (6.07%)
Summation T_I_	69.68 ± 0.44	54.81 ± 0.14 (7.93%)		56.76 ± 0.10 (6.85%)
Addition T_R_	51.14 ± 0.16		45.47 ± 0.03 (8.03%)	44.63 ± 0.07 (5.48%)
Summation T_R_	57.49 ± 0.29		51.96 ± 0.04 (10.80%)	53.35 ± 0.11 (8.19%)

Accuracy data suggests the MODWT provided better accuracy particularly for the return (T_R_) compared to the intial (T_I_) study period, where the former is characterized by series volatility. Reported are mean ± standard error mean absolute percentage error (MAPE), ARIMA, autoregressive integrated moving average; GARCH, generalized autoregressive conditional heteroscedasticity; MODWT, maximum overlap discrete wavelet transform.

## 4. Discussion

Since the early descriptions of equations of learning theory by Ebbinghaus (1880), performance has been considered as smooth decelerating functions of time (Jaber, 2019). Subsequently, early behavioral theories were formulated, championed by Edward Thorndike, where learning was thought to be governed by three laws (effect, readiness, and exercise), and it was believed that learning occurred as a consequence of positive and negative associations between stimuli and responses (Thorndike, 1913). In the 1950's mathematical theories of learning focused on formulating predictive equations for behavioral responses, particularly in probability space (Howard, 2014). In previous work, it was demonstrated that by capturing variation in performance using an ARIMA model, forecast accuracy in mental arithmetic using the soroban could be improved over models that are limited to estimating mean response time only. Additionally, predictions by an ARIMA model showed no statistically significant difference from the actual test values (Abdul-Rahman, 2020). In the current empirical study, performance modeling was extended into the frequency domain where forecast accuracy was shown to be more favorable compared to the corresponding time-domain approaches. The reason behind these analytic gains is likely due to smoothing and noise reduction by frequency decomposition, which may have improved predictive accuracy (Cohen, 2014). The other advantage of frequency decomposition is that the evolution of the time series can be observed and quantified. Additionally, the phase of the time series of the two tasks can be accurately estimated and in our case becomes similar in the return study period between the two tasks. Mental arithmetic task-dependent differences in cognitive processing may underlie these within period response time and phase differences. Of the various models of cognitive arithmetic processing, they all share common assumptions, where performance on simple arithmetic operations depends on retrieval from long-term memory; the memory representation is organized and structured in terms of the strength of individual connections and reflects varying degrees of relatedness among the elements; and the strength with which the elements are stored. Hence the probability or speed of retrieving information depends critically on experience, especially acquisition, rather than on numerical characteristics inherent in the information itself (Ashcraft, 1992). Additionally, when comparing 20% of each at the end of the initial and first 20% of the return study periods, there was a loss of learning gain, however, the loss was minimal (3.52 s for addition and 6.26 s for summation). It is likely the influence of the abacus-based visuospatial imagery format on long-term memory may have helped to mitigate the extent of skill degradation and retained this at a minimum level. Moreover, studies on fluency in addition and summation conclude that subtraction is a more difficult task compared to addition as subjects deduce differences from their knowledge of sums (Kamii et al., 2001). This interpretation is assumed in the light of Piaget's theory where a developmental characteristic is the general primacy of the positive aspect of actions, perception, and cognition over the negative aspect (Piaget, 1976). Therefore, differences in response time are thought to be dependent on differences in long-term memory retrieval, particularly in associations between nodes of memory networks (Ashcraft, 1992). It is interesting to note, that this differs from a cognitive perspective and extends beyond memory-based responses to spatial interactions. Current theories of arithmetic processing make different predictions for the interaction between space and mental arithmetic, these studies tested the hypothesis that addition and summation can cause shifts of spatial attention, where right-side targets are detected faster than left-side targets when preceded by an addition operation, and left-side targets are detected faster than right-side targets when preceded by a subtraction operation, these findings have been replicated in several studies (Li et al., 2018). This evidence suggests that cognitive mechanisms that underlie the differences in mental arithmetic processing may extend beyond long-term memory retrieval only or the presence of operation-dependent neural pathway interactions occur during the retrieval process.

All models demonstrated a reduction of response time with learning for both mental arithmetic tasks, more notable during the initial study period for the addition task. The visuospatial format of numerical representations in the soroban likely enhanced these learning gains, where computations are represented as a function of the changing bead configurations. Hatano et al. examined representational changes in digit memory as a function of expertise in mental-abacus operation in five groups of 54 operators differing in skill. They reported an inverse relationship between operator skill and memory vulnerability for digits to an aural-verbal interpolated task and a direct relation to the vulnerability to an interpolated visual-spatial task, which suggests that advanced operators apply the mental abacus calculations to visual memory (Hatano et al., 1987). These results were confirmed by Frank and Barner who in a study on 38 subjects speculated that numerical computations were represented in the visual working memory by splitting the abacus into a series of columns, each of which they thought was independently stored as a unit with its own detailed substructure (Frank and Barner, 2012). Most individuals acquire this ability after abacus skills have become automated, and possess the ability to mentally manipulate the abacus synonymous with operating a real abacus (Stigler, 1984). This dependence on visual imagery may allow a rapid progression in the learning task by utilizing alternative analytic resources and minimizing skill degradation. Researchers have also argued that additional cognitive advantages of abacus training may involve multiple components of working memory and exert a transfer effect, thereby improving visuospatial memory for other tasks (Wang, 2020). Comparisons of visuospatial memory span tests between subjects skilled in abacus-based mental arithmetic techniques and those who lack this skill demonstrated significant performance differences in both cross-sectional and longitudinal study designs (Bhaskaran et al., 2006; Chen et al., 2006; Lee et al., 2007; Kamali et al., 2019). These visuospatial and working memory effects are likely to have acted to improve computational skills during task learning in the current empirical study. Due to the auditory nature of the presented stimulus, further learning gains would have been achieved through the phonological loop, which refers to the temporary storage of phonological and auditory information (Wang, 2020). Although untested in the current study, higher digital spans have been found in subjects skilled in abacus-based mental arithmetic methods compared to those in standard arithmetic skills. Hatano and Osawa reported a larger than average digit span in three abacus-based mental arithmetic experts. This finding was specific for digits but not object names or letters. Additionally, they reported that the digit span of abacus experts was more affected by concurrent visuospatial distractors in contrast the digit span of non-experts was more affected by concurrent verbal distractors (Hatano and Osawa, 1983). Similarly, Hatta et al. investigated digit spans in 29 soroban experts they reported a superior ability in digit memory in soroban experts, digital memorization competence of soroban experts was reduced by the presence of pictorial soroban figures but was not reduced by the presentation of digits, seems to indicate that experts utilize images which are analogous to the actual soroban as an aid to hold numbers in memory. Moreover, soroban experts were noted to make special types of error, such as the number five error, more than control subjects, this also seems to suggest that soroban experts utilize soroban images; as frequent occurrences of errors of this type can only be explained by soroban visualization (Hatta et al., 1989). Recently, the neurophysiological mechanism underlying the superior short-term memory for digits in abacus experts was investigated by Tanaka et al. in a functional magnetic resonance imaging study. They compared the brain activity of abacus experts and non-experts during the memory retention period. They found that whereas in controls, activity was greater in cortical areas related to verbal working memory, including Broca's area, in experts, activity was greater in cortical areas related to visuospatial working memory, including the bilateral superior frontal sulcus and superior parietal lobule (Tanaka et al., 2002). Therefore the visuospatial and the phonological loop properties of abacus-based operations have unique advantages on the working memory and may consequently allow a longer-term higher skill retention.

In general, the differences in time, and frequency domain properties of all time series may be attributable to different properties of the learning process described in what is known as phase theories, where initial, intermediate, and established phases of learning exhibit different relationships between knowledge structures, where ultimately a level of automaticity is achieved (Shuell, 1990). These differences in performance over time generate analytic challenges, where non-stationarity, heteroscedasticity, and non-normal distribution of test variables were the most prominent. Non-stationary frequency characteristics of the series favored frequency decomposition methods other than Fourier analysis, as classical assumptions do not apply in the presence of non-stationarity, non-periodicity, and signal volatility. Continuous Wavelet transform decomposed the time series into a linear combination of different frequencies, and therefore was able to capture dynamics in period and intensity and to model the trend in the series. Although there were no specific localizing features in the power spectra, it provided an overview of the signal of the series and phase relationships. It allowed the exact frequencies at which dominant learning across the entire time series, thereby enabling direct visualization of the evolution and volatility of the learning process throughout the series even in the presence of non-stationary frequency characteristics (Schlüter and Deuschle, 2010; Avdeeva et al., 2021). In the current study, phase differences were minimal in the T_*R*_ period, this probably indicates stabilization of the learning process to a constant low rate between the two tasks. The multi-resolution decomposition wavelet transformation allowed for frequency and scale-specific variance of the series, therefore forecast accuracy using wavelet transformation which addressed these barriers was more favorable (MAPE 5.48–8.19%) than the strictly time-domain models, where forecast accuracy was degraded (MAPE 6.16–10.80%), especially in the presence of volatility. Recently Pathan et al. (2019) described the use of a DWT in the classification of efficient mental arithmetic tasks using Functional Near-Infrared Spectroscopy an alternative to EEG, they reported an accuracy of 93.26% using DWT-based features in a support vector machine algorithm. Karthikeyan et al. used a DWT to detect autonomic nervous system activity generated from stress response to mental arithmetic, they reported a classification rate of 96.3 and 75.9% in low and high-frequency bands respectively (Karthikeyan et al., 2012). Several studies suggest that ARCH/GARCH models outperform ARIMA models in short-term forecast accuracy and asymmetric heavy-tailed distributions (Sparks and Yurova, 2006; Ekinci, 2021). Heteroscedasticity (non-constant variance of the error term) appeared in the T_*R*_ time series, which has a known degrading influence on model forecast accuracy (Wang and Akabay, 1994; Schlüter and Deuschle, 2010). Heteroscedasticity was likely due to the number of complementary operations in a single calculation, a property that was not taken into account in the models resulting in variable performance.

The non-normal distribution of performance times is consistent with the distribution of response times predicted in Item Response Theory. In an analysis of reading speed Rasch derived a gamma distribution for the response time, and a Poisson distribution for the number of items completed, this is known as the Rasch model (Rasch, 1993). Although the difference in performance time between correct and erroneous responses contracted in the T_*R*_ period by 20–40% (from 5 to 4 s for the summation task, and 5–2 s for the addition task), performance time was consistently longer for tasks with errors, the difference remained statistically significant throughout the test period. In a population of 894 subjects, Lasry et al. reported a difference of approximately 50% between correct and erroneous responses to conceptual questions (Lasry et al., 2013). This is known as the speed-accuracy trade-off, where errors are more probable with longer task performance times (van der Linden, 2007; Lasry et al., 2013).

The limitation of wavelet transform is an increase in the model complexity. Additionally, the non-standardization of wavelet choice from an extended family of basis functions introduces ambiguity in the optimal approach. However, time-series characteristics like volatility or the existence of long-term trends and the forecasting horizon are the most significant factors influencing forecast accuracy and are addressed appropriately by wavelet transformation (Schlüter and Deuschle, 2010).

## 5. Conclusion

The time and frequency properties of learning response times are useful to consider in understanding learning processes. Wavelet transformation is a useful method of time series decomposition where variables are non-stationary, heteroscedastic, and non-normal. Wavelets can characterize changes in the evolution and phase of the series in addition to generating accurate forecasts from this method compared to strictly time-domain models.

## Data availability statement

The original contributions presented in the study are included in the article/Supplementary material, further inquiries can be directed to the corresponding author.

## Ethics statement

Ethical review and approval was not required for the study on human participants in accordance with the local legislation and institutional requirements. Written informed consent for participation was not required for this study in accordance with the national legislation and the institutional requirements. Written informed consent was obtained from the individual(s) for the publication of any potentially identifiable images or data included in this article.

## Author contributions

AA-R conceived and designed of the study, organized the database, performed the statistical analysis, developed the R code, and wrote the manuscript.

## Conflict of interest

The author declares that the research was conducted in the absence of any commercial or financial relationships that could be construed as a potential conflict of interest.

## Publisher's note

All claims expressed in this article are solely those of the authors and do not necessarily represent those of their affiliated organizations, or those of the publisher, the editors and the reviewers. Any product that may be evaluated in this article, or claim that may be made by its manufacturer, is not guaranteed or endorsed by the publisher.
